# Adults with intellectual disabilities’ satisfaction regarding their hospitalization: A correlational descriptive study

**DOI:** 10.1177/17446295231193461

**Published:** 2023-08-11

**Authors:** Morgane Gilliand, Ariane Bernier Emch, Béatrice Perrenoud

**Affiliations:** HESAV School of Health Sciences, 87680 University of Applied Sciences and Arts Western Switzerland (HES-SO), Lausanne, Switzerland; Institute of Higher Education and Research in Healthcare-IUFRS, Lausanne University Hospital, University of Lausanne, Lausanne, Switzerland; 31128 Clinical Nurse Specialist, Lavigny Institute, Lavigny, Switzerland; Lausanne University Hospital (CHUV), Lausanne, Switzerland; La Source School of Nursing Sciences, University of Applied Sciences and Arts Western Switzerland (HES-SO), Lausanne, Switzerland

**Keywords:** adults, cognitive appraisal, hospitalization, intellectual disability, satisfaction

## Abstract

When hospitalized, adults with intellectual disabilities are more anxious and have more unmet needs than the general population. Despite these problems, studies report contradictory results about their satisfaction with hospitalization. The aim of this study was to determine the level of satisfaction of adults with intellectual disabilities regarding their hospital care and the factors associated with satisfaction. An analysis of the Patient Satisfaction Scale (PSS) and Cognitive Appraisal of Health Scale (CAHS) instruments completed by adults with intellectual disabilities, or their caregivers, after hospitalization was done. The 32 participants’ mean PSS score was 3.6/5, with means of 13.3/25 and 8.7/25 on the CAHS’ ‘harm/loss’ dimension and ‘challenge’ dimension, respectively. None of the factors studied was associated with the total PSS score. Adults with intellectual disabilities were not fully satisfied with their hospital care, experiencing challenges and losses. These findings call for a rethink of the care provided to this population.

## Introduction

Adults with intellectual disabilities are in poorer physical and mental health than the general population (GP) (Reppermund et al., 2017; Robertson et al., 2015). Adults with intellectual disability, for example, are more likely to have diabetes, epilepsy, gastrointestinal problems or anxiety than the GP (Robertson et al., 2015). They are therefore hospitalized more often, with longer lengths of stay (LOS) (Iacono et al., 2020; Skorpen et al., 2016; Weise et al., 2021), and have more unmet needs during those hospitalizations (Iacono et al., 2014; McCormick et al., 2021). Adults with intellectual disabilities are also more likely to have undiagnosed conditions, to be misdiagnosed, and to experience adverse events (Ailey et al., 2015; Mimmo et al., 2022): they therefore frequently experience health inequalities (Mimmo et al., 2022; Schalock et al., 2021).

Hospitalization may be a source of fear, stress and anxiety for some adults with intellectual disabilities (Drozd et al., 2020; Iacono et al., 2014) as healthcare systems are ill-equipped to meet their needs (Mimmo et al., 2022): they have difficulty adapting to new situations (Drozd et al., 2021) and are more vulnerable to anxiety than the GP because of their limited supports and low level of education (Hermans et al., 2014). Adults with intellectual disabilities reported problems in their interactions with healthcare professionals (Tuffrey-Wijne et al., 2014; Drozd et al., 2020, 2021). Adults with intellectual disabilities also report facing negative attitudes (Iacono et al., 2014; McCormick et al., 2021) and not being adequately informed about their diagnoses, procedures, and treatments (Ali et al., 2013; McCormick et al., 2021), even when supported by their caregiver (Charles, 2020; Ninnoni, 2019). Adults with intellectual disabilities also report that the information provided is rarely understandable to them (Ali et al., 2013; Ninnoni, 2019; McCormick et al., 2021).

Healthcare professionals report having limited knowledge and skills for caring for adults with intellectual disabilities (Charles, 2020; Iacono et al., 2014), which leads to workplace stress and anxiety (Pelleboer-Gunnink et al., 2017). They encounter problems with communication and the transmission of information (Ali et al., 2013; Ndengeyingoma and Ruel, 2016). They therefore prefer to address caregivers directly because they find this more efficient (Charles, 2020; Hemsley and Balandin, 2014).

**Figure 1.**
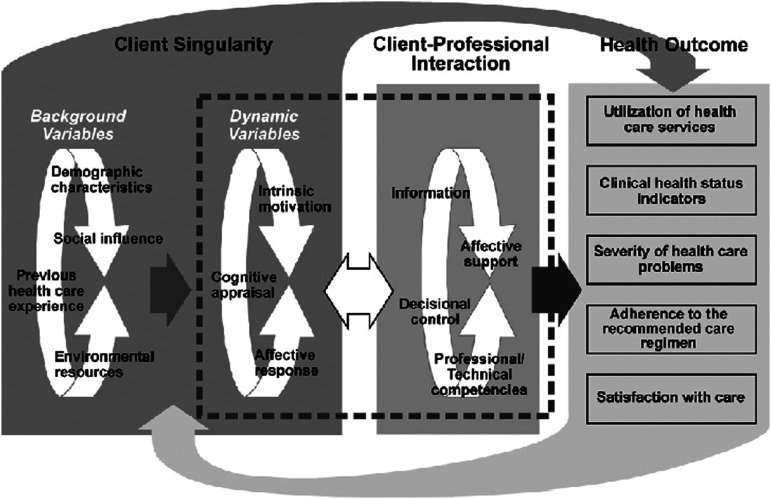
Cox’s Interaction Model of Client Health Behavior (Cox, 2003).

According to Cox’s Interaction Model of Client Health Behavior, client singularity (a person’s unique personal background and context) influences client–professional interactions and the client’s satisfaction with the care received (Figure 1). Client singularity considers sociodemographic, clinical, and hospitalization data plus a cognitive appraisal (Cox, 2003), defined as the process by which people evaluate a potentially stressful event for its significance and importance to their own well-being (Ahmad, 2005; Cox, 2003). Client–professional interaction refers to the therapeutic content and process that takes place between a patient and a healthcare professional (Cox, 2003). Satisfaction is a subjective multidimensional concept and an important indicator of the quality of care (Levandovski et al., 2015; Santos de Almeida et al., 2015). Satisfaction with care is defined as a measure of the patient’s or the family’s opinions of the care received from nursing staff (Cox, 2003). Cox (2003) stated that “although satisfaction with care is not a behavioral measure, it is a strong indicator of subsequent behavior” (p. 97).

Studies including adults with intellectual disabilities report conflicting results about satisfaction regarding their hospitalization (Iacono and Davis, 2003; Vogan et al., 2017). However, given the problems adults with intellectual disabilities encounter, and according to the relationships established in Cox’s model, adults with intellectual disabilities tend to be dissatisfied with their hospital care. Dissatisfaction can, nevertheless, have a major influence on adults with intellectual disabilities’ future health because “satisfaction with care is linked very closely to whether patients follow a therapeutic protocol and implement suggested health behaviors” (p. 97) (Cox, 2003). In addition, measuring patient satisfaction provides healthcare professionals with feedback that allows them to reorganize care processes with regards to the client’s needs and the institution’s objectives, thus improving the quality of care provided (Santos de Almeida et al., 2015). Exploring adults with intellectual disabilities’ satisfaction regarding their hospitalization can, therefore, help to reduce the health inequalities affecting this population (Mimmo et al., 2022; Schalock et al., 2021).

In the Cox model, sociodemographic, clinical, and hospitalization data, together with cognitive appraisal, are all associated with hospital satisfaction (Cox, 2003). The literature has notably shown that age (Findik et al., 2010), LOS (Levandovski et al., 2015), and healthcare professionals’ consideration of specific needs (Ali et al., 2013) are associated with the satisfaction of adults with intellectual disabilities. Communication disorders also influence satisfaction (Buzio et al., 2002). Adults with intellectual disabilities are not always able or dare to express their needs in hospital (Iacono and Davis, 2003), which is why they report needing the presence of their caregiver (Drozd et al., 2021; Iacono and Davis, 2003). Indeed, a caregiver’s presence is positively associated with adults with intellectual disabilities’ satisfaction during hospitalization (Ali et al., 2013; Iacono et al., 2014). However, caregivers report difficulties having their expertise recognized by healthcare professionals (Ali et al., 2013; Hemsley and Balandin, 2014).

**Figure 2.**
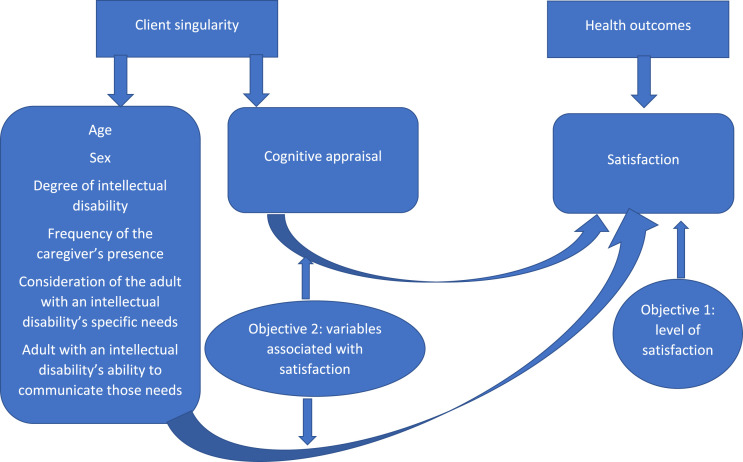
Adaptation of the Cox model to the present study.

The present study’s primary objective was to determine adults with intellectual disabilities’ levels of satisfaction with their nursing care during a somatic care hospitalization. Its secondary objective was to determine whether their cognitive appraisal, hospitalization, sociodemographic or clinical data were associated with their satisfaction, as hypothesized in the Cox model (Figure 2).

## Method

### Participants

This cross-sectional, descriptive correlational study included a convenience sample of adults with intellectual disabilities. However, caregivers (family members or social services professionals) could respond in place of that person if they were unable to respond alone.

Inclusion criteria for adults with intellectual disabilities were being: a) aged 18 years old or more; b) diagnosed with an intellectual disability (or any synonym of intellectual disability, including developmental disabilities, mental retardation, mental infirmity, or learning disabilities); and c) hospitalized for at least one day and one night in a somatic care setting in the last two months in the French-speaking part of Switzerland. The presence of intellectual disability was self-reported by the individual or their family or professional caregiver. In cases of multiple hospitalizations, participants were asked to just consider the most recent one. Exclusion criteria were not understanding French or hospitalization in psychiatric care.

Inclusion criteria for family or professional caregivers were: a) being aged 18 years old or more; b) having provided care, for at least one year, to an adult with an intellectual disability who met the inclusion criteria; and c) being the caregiver of an adult with little or no verbal communication ability. The caregiver also needed to be present every day for hospitalizations of 48 hours or less and at least three times for longer hospitalizations. Exclusion criteria for family or professional caregivers were not understanding, reading, or speaking French.

### Procedure

Recruitment took place between September 2018 and February 2019 via ten institutions providing care and support for adults with intellectual disabilities and two associations for the families of people with intellectual disabilities in the Swiss cantons of Vaud, Fribourg, and Neuchâtel. Family or professional caregivers were informed of the study by email. People with intellectual disability were informed by the person in charge of the study in the institution or by the family caregiver. When an adult with intellectual disabilities wanted to participate, the investigators also introduced their project to people with intellectual disabilities and obtain his/her final consent. During the study, follow-ups with participants occurred every month via the institutions and once via the associations. Potential participants wishing to be involved contacted the investigators, who then investigated whether they met the inclusion criteria. If the adult with an intellectual disability declared that they could read and write, they completed the questionnaire independently. If they had good comprehension and communication skills but were unable to complete the questionnaire alone, one of the investigators met with them, read them all the questions, and recorded their answers. If they had little or no verbal communication skills, the questionnaire was completed by the family or professional caregiver. The professional or family caregiver was led to hetero-represent the person with an intellectual disability not to share his/her own experience of hospitalization. This choice was made because professional and family caregivers are the best representatives we had for this target audience (Iacono and Davis, 2003).

### Measurement instruments

Adults with intellectual disabilities’ satisfaction with hospital care was measure using the Patient Satisfaction Scale (PSS) developed by Risser in 1975 (Levandovski et al., 2015). It consists of 25 questions assessing three dimensions of nursing care using a Likert scale ranging from 1 (strongly disagree) to 5 (strongly agree). The three dimensions are: ‘technical/ professional activities’, including knowledge and skills (seven items); ‘educational relationship’, referring to nurses’ ability to convey appropriate information (seven items); and ‘relationship’, regarding trusting relationships that enable constructive patient–nurse interaction (11 items). To calculate mean scores, scores for negative items were reversed (Levandovski et al., 2015). This instrument was adapted to assess satisfaction with inpatient and outpatient settings (Rose, 2010), and it demonstrates moderate-to-strong internal consistency, with Cronbach’s alphas between 0.78 and 0.88 in its English version (Rose, 2010) and greater than 0.7 in its Greek version (Charalambous and Adamakidou, 2012). The higher the PSS score, the more satisfied the patient was (Levandovski et al., 2015).

Other patient variables in our study were a cognitive appraisal and secondary personal data. Cognitive appraisal evaluates an individual’s response to a stressful event—in this case, their hospitalization—and this was done using the Cognitive Appraisal of Health Scale (CAHS) developed by Kessler in 1998. The scale has been validated with prostate cancer patients. It is composed of 13 items assessing three dimensions using a Likert scale ranging from 1 (strongly disagree) to 5 (strongly agree). The three dimensions are: ‘threat’, which describes the harm or loss anticipated by the patients but which has not yet occurred (five items); ‘damage/loss’, which describes harm that has already occurred, as perceived by patients (five items); and ‘challenge’, which involves a patient’s judgment made to overcome an event that may be stressful (three items). The CAHS demonstrates moderate-to-strong internal consistency, with a total Cronbach’s alpha of 0.70; and alphas of 0.79 for ‘damage/loss’, 0.74 for ‘threat’, and 0.70 for ‘challenge’ (Ahmad, 2005). Scores are interpreted by dimension. The present study did not consider the dimension of ‘threat’ as losses could not be anticipated—the hospitalization was over. High scores for the ‘damage/loss’ dimension indicate that adults with intellectual disabilities had a poor experience of hospitalization. High scores for the ‘challenge’ dimension indicate that people with intellectual disabilities did not experience their hospitalization as a challenge (Ahmad, 2005). The secondary data examined includes:

- Sociodemographic: sex (men or women) and age (in years)
- Clinical: self-reported degree of intellectual disability (mild, moderate, severe and profound), self-reported ability of adults with intellectual disabilities to communicate their needs (Likert in 5 points: from never to always),
- Hospitalization data: LOS (in day), presence of a caregiver (Likert in 5 points: from never to always), self-reported consideration of adults with intellectual disabilities’ specific needs by healthcare professionals (Likert in 5 points: from never to always).

These data were collected using a questionnaire developed for this study.

The PSS and CAHS were translated into French using the recommended back-translation method (Polit and Beck, 2017). The investigators also ensured that the translation was culturally appropriate to the participants. In addition, the French version of the questionnaire was reviewed for sentence structure, length, and the simplicity of the terms used, thus ensuring optimal readability and comprehension.

As the PSS and CAHS have been translated and to our knowledge have never been used with adults with intellectual disabilities and their family or professional caregivers, a pretest was individually conducted with five adults with intellectual disabilities and five family or professional caregivers using the ‘think aloud’ method (Hauge et al., 2015). This method collects people’s thoughts about their understanding of the questionnaire. During the pre-tests, the questions were addressed orally to the five persons with an intellectual disability and to the five family caregivers, by the investigators. The investigators then asked the person about his/her understanding. In order to avoid the acquiescence bias that is very common among the target audience, the investigators asked the persons with intellectual disabilities to rephrase the question in their own words. Because the pretest did not result in any changes, the data were used in this article’s analysis.

### Ethical considerations

This study was approved by the Cantonal Commission for Ethics in Human Research of the Canton of Vaud in Switzerland (project number CER-VD:2018-01332). Consent was obtained from adults with intellectual disabilities capable of discernment or their legal representatives if they were incapable. In addition, to respect the self-determination of people with intellectual disabilities, oral or written assent was requested from all persons with intellectual disabilities who were incapable of discernment but had the capacity to participate in this study. All data were coded and treated as confidential. The investigators—nurses experienced in caring for adults with intellectual disabilities—were attentive to participants’ functioning and capacities so that interviews could occur in optimal conditions (with a caregiver present, breaks if necessary, and the opportunity to answer questions part by part).

### Data analysis

Statistical analyses were performed using Stata software (version 15.1). A two-sided alpha significance level of 0.05 was used. Descriptive analyses were performed on all variables. Kendall’s tests (Tau–b) were performed between satisfaction data (PSS total scores; PSS dimension scores) and secondary data or participants’ cognitive appraisals of their hospitalization. The small sample size required an analysis of medians; however, means were also used to allow comparison with published results.

## Results

### Sample characteristics

Thirty-two adults with intellectual disabilities participated in the present study (Table 1), 66% of them were hetero represented by a family or professionals’ caregiver. Fifty-three percent of the sample were men. Mean participant age was 48.7 years old and the mean hospital LOS was 8.8 days. Just over half (56%) had severe or profound intellectual disabilities, and 67% had difficulty communicating their needs to healthcare professionals. Most caregivers (72%) were often or continuously present during the hospitalization. Just over half of the participants (53%) communicated that their needs as an adult with intellectual disabilities were properly taken into account by healthcare professionals. The mean scores on the CAHS were 13.3/25 for the ‘damage/loss’ dimension and 8.7/15 for the ‘challenge’ dimension.

**Table 1.** Participants’ characteristics (N = 32).

	n (%)	M (SD); Med (IQR); Min–Max
Participants		
Auto represented adult with intellectual disabilities	11 (34.4)	
Hetero represented adult with intellectual disabilities	21 (65.6)	
Men	17 (53.1)	
Age (years)		48.7 (15.7); 49.0 (24.5); 19.0–76.0
Hospital LOS (days)		8.8 (5.8);7.0 (4.5); 2.0–28.0
Degree of intellectual disability		
Light	5 (15.6)	
Moderate	9 (28.1)	
Severe	7 (21.9)	
Profound	11 (34.4)	
Adult with intellectual disability’s ability to communicate their needs		
Never	3 (9.4)	
Rarely	12 (37.5)	
Sometimes	7 (21.9)	
Often	3 (9.4)	
Always	7 (21.9)	
Frequency of caregiver presence		
Never	1 (3.1)	
Rarely	2 (6.3)	
Sometimes	6 (18.8)	
Often	11(34.4)	
Always	12 (37.5)	
Participants’ perceived frequency with which healthcare professionals considered their specific needs		
Never	0	
Rarely	3 (9.4)	
Sometimes	12 (37.5)	
Often	10 (31.3)	
Always	7 (21.9)	
CAHS		
Dimension: Damage/Loss		13.3 (5.1); 12.0 (6.5); 5.0–25.0
Dimension: Challenge		8.7 (2.5); 9.0 (3.0); 3.0–15.0

*n* = number; *M* = mean; *SD* = standard deviation; Med = median; IQR = interquartile range; Min = minimum; Max = maximum.

### Adults with intellectual disabilities’ satisfaction scores for nursing care received in hospital

Participants had a mean overall score of 3.6/5 on the PSS (*SD* = 0.6; *Med* = 3.7). Regarding the PSS’s dimensions, participants had mean scores of 3.9/5 for ‘technical/occupational activities’ (*SD* = 0.7; *Med* = 3.9), 3.2/5 for ‘educational relationship’ (*SD* = 0.6; *Med* = 3.1), and 3.8/5 for ‘relationship’ (*SD* = 0.7; *Med* = 4.0).

### Variables associated with hospital satisfaction among adults with intellectual disabilities

Age, hospital LOS, degree of intellectual disability, the adult with an intellectual disability’s ability to communicate their needs, frequency of a caregiver’s presence, and consideration of the specific needs of adults with intellectual disabilities were not correlated with participants’ satisfaction scores (*p* > 0.05) (Table 2). The scores obtained in the CAHS’s ‘challenge’ and ‘damage/loss’ dimensions were also uncorrelated with participants’ satisfaction scores (*p* > 0.05).

**Table 2.** Correlations between PSS scores and secondary variables.

PSS factors of influence	PSS: Total		PSS: Educational relationship		PSS: Educational relationship		PSS: Relation	
	τ	p	τ	p	τ	p	τ	p
Age	0.0	0.83	0.0	0.73	-0.2	0.21	0.0	0.88
Length of stay	-0.1	0.69	-0.2	0.24	0.0	0.97	0.0	0.90
Degree of intellectual disability	0.2	0.10	0.1	0.54	0.2	0.25	0.3	0.04*
Adult with intellectual disability’s ability to communicate their needs	-0.2	0.22	0.0	0.79	-0.1	0.42	-0.2	0.10
Frequency of caregiver presence	0.2	0.25	0.1	0.67	0.2	0.20	0.1	0.34
Participants’ perceived frequency with which healthcare professionals considered the specific needs of adults with intellectual disability	0.2	0.14	0.3	0.03*	0.1	0.47	0.1	0.30
CAHS: Challenge	0.0	0.88	0.1	0.55	0.1	0.65	-0.1	0.59
CAHS: Damage/ Loss	-0.2	0.13	-0.3	0.02*	-0.1	0.69	-0.2	0.22

τ = Kendall’s test, tau-b; p = p value.

* = significant.

The tests did show a statistically significant positive correlation between scores for the PSS’s ‘technical/work activities’ dimension and participants’ perceptions of healthcare professionals’ consideration of their needs (*p* = 0.03) (Table 2): i.e., the more adults with intellectual disabilities perceived that health professionals were considering their specific needs, the more satisfied they were with nurses’ technical and professional interventions. There was a statistically significant negative correlation between scores on this ‘technical/work activities’ dimension and scores on the CAHS’s ‘damage/loss’ dimension (*p* = 0.02): i.e., the more damages and losses adults with intellectual disabilities experienced, the less satisfied they were with the care they received. A statistically significant positive correlation was also observed between scores on the PSS’s ‘relationship’ dimension and the degree of intellectual disability (*p* = 0.04): i.e., the higher the level of intellectual disability, the more satisfied participants were with their relationships with the nurses. The other influencing factors studied did not correlate with scores for the PSS’s dimensions (*p* > 0.05).

## Discussion

Exploring adults with intellectual disabilities’ satisfaction with their hospitalization and the factors associated with their satisfaction can help reduce the health inequalities experienced by this population (Mimmo et al., 2022; Schalock et al., 2021) by enabling us to identify actions that can be taken to improve their care.

### Sample characteristics

Our study’s male/female distribution was comparable to that of other studies dealing with the satisfaction of adults with intellectual disabilities (Iacono and Davis, 2003; Vogan et al., 2017) and to their distributions in Swiss institutions for the disabled (Swiss Federal Statistical Office, 2022), from which almost all of our sample came (97%). This distribution is not, however, representative of the GP of adults with intellectual disabilities, which includes more men than women (McBride et al., 2021; Schalock et al., 2021).

In addition, the distribution of disability levels is not representative of the GP of adults with intellectual disabilities, which includes more mildly and moderately disabled than severely and profoundly disabled individuals (McBride et al., 2021; Schalock et al., 2021). The low representativeness of adults with mild or moderate intellectual disabilities can be explained by the fact that few of our participants lived at home. Indeed, it is likely that people living at home have milder degrees of intellectual disability than people living in institutions.

Our sample’s adults with intellectual disabilities also had longer average hospital LOS than Switzerland’s GP. In 2020, the average hospital LOS in Switzerland was 5.3 days (Swiss Health Observatory, 2022), so our sample’s participants were hospitalized for an average of 3.5 days longer than Switzerland’s GP. This difference has been demonstrated in other studies (Iacono et al., 2020; Skorpen et al., 2016; Weise et al., 2021) and might be explained by the fact that adults with intellectual disabilities are less healthy than the GP (Ndengeyingoma and Ruel, 2016; Reppermund et al., 2017; Robertson et al., 2015) and that caring for them is very complex for healthcare professionals (Charles, 2020; Iacono et al., 2014).

Regarding cognitive appraisals, our study’s results suggest that adults with intellectual disabilities face cognitive adaptation difficulties during hospitalization because they experience losses and challenges. To the best of our knowledge, there are no data available with which to compare these results, as we found no other quantitative studies on the cognitive appraisal of adults with intellectual disabilities. Various qualitative studies have, nevertheless, reported that hospitalization is indeed a difficult experience for adults with intellectual disabilities (Hemsley and Balandin, 2014; Drozd et al., 2020), because they have difficulty adapting to new situations (Drozd et al., 2021).

### Adults with intellectual disabilities’ satisfaction scores for their hospital nursing care

Our study’s results suggested that adults with intellectual disability were not fully satisfied with the care patients received from nurses in hospital. They also suggested that they were not fully satisfied with the hospital care overall because measuring satisfaction with nursing care is a reliable representation of overall hospital satisfaction (Levandovski et al., 2015).

Our sample’s mean satisfaction score was close to that of a study in Canada by Vogan et al. (2017) (M = 3.78). However, in their study Vogan and al. (2017) did not use a valid tool to measure satisfaction (for each service used, participants were asked to rank their satisfaction with the service on a scale from 1 (very dissatisfied) to 5 (very satisfied) and an average satisfaction score was computed for each participant, focuses on a specific population (40 people with a diagnosis of Asperger Syndrome with and without intellectual disability), and includes satisfaction with different types of care services, not just hospitalizations (e.g., family physician, dentist) (p. 3).

Our participants were, however, more satisfied with the care they received than Australian people in the study of Iacono and Davis (2003) (*M* = 2.9/5). This study did not use a validated instrument to measure the satisfaction of adults with intellectual disabilities (questionnaire developed for the purpose of the study and interviews). In their study Iacono and Davis (2003) included 328 people among which “223 people had an intellectual disability and 238 had a physical disability, such that they used a wheelchair or needed help with mobility” (p.254). Moreover in general, Switzerland’s patients are more satisfied with their healthcare system’s functioning than Australia’s (Swiss Confederation, 2016), which could explain the difference in results. It could also be explained by the inclusion of emergency department patients in Iacono and Davis’ (2003) study. Emergency department care is not comparable to hospital care, because emergency care visits can be extremely stressful (Tint et al., 2019).

Our participants were nevertheless less satisfied with their care than adults with intellectual disabilities (N = 30) hospitalized at Geneva University Hospitals, Switzerland (*M* = 3.6/5 vs *M* = 4.7/5). In this project, satisfaction was evaluated with a question using a 5-point Likert scale (from no, not at all to yes, completely), which may explain the difference in results. This particular difference in results could also be explained by the fact that Geneva has set up a specific support program for people with intellectual disabilities (Lalive d’Epinay Raemy and Paignon, 2019).

Our sample’s adults with intellectual disabilities were also less satisfied with the quality of the care they received than was Switzerland’s GP, as represented by individuals who participated in a 2019 survey (*M* = 4.2/5) (National Association for Quality Development in Hospitals, 2019). This difference can also be explained by the fact that in the National Association for Quality Development in Hospitals (2019) satisfaction was assessed with a question and a 5-point Likert (from 1 = most negative response to 5 = most positive response). This difference in satisfaction could also be related to the communication disorders present among our sample’s adults with intellectual disabilities. Communication disorders are negatively correlated with hospital satisfaction (Buzio et al., 2002).

### Variables associated with adults with intellectual disabilities’ hospital satisfaction

The sociodemographic, clinical, and hospitalization factors in our study were not associated with adults with intellectual disabilities’ overall mean satisfaction score. However, other studies have reported that age (Findik et al., 2010), LOS (Levandovski et al., 2015), and frequency of caregiver presence (Ali et al., 2013; Iacono et al., 2014) were associated with satisfaction. Communication disorders also had a negative influence on satisfaction (Buzio et al., 2002). These differences could be explained by our sample’s small size and relatively large number of individuals with communication problems.

In our sample, the degree of intellectual disability was positively correlated with the score on the PSS’s ‘relationship’ dimension, suggesting that the more severe the disability, the more satisfied those adults were with their interactions with nurses. This result is surprising, however, because the degree of intellectual disability affects a person’s ability to communicate (Schalock et al., 2021) and should, therefore, make interactions with nurses more complicated. This finding suggested that caregivers may have underestimated the difficulties that adults with intellectual disabilities experience in interactions, although no correlation was observed between our participants’ satisfaction and their abilities to communicate their needs. Scott and Havercamp (2018) reports that caregivers have a poorer perception of health-related factors than people with intellectual disability, especially when they are subjective. This was the case for most of the adults with intellectual disabilities participating in our study (84%). The other possible explanation is that caregivers have different expectations than people with intellectual disabilities, a difference in perception that Iacono and Davis (2003) also highlighted. Their study showed that only a minority of participants were satisfied with their care, whereas caregivers were somewhat satisfied with the care provided (Iacono and Davis, 2003). The difference in perception between people with intellectual disabilities and caregivers could be explained by the fact that healthcare professionals communicate mainly with caregivers (Hemsley and Balandin, 2014; McCormick et al., 2021). The present study’s great involvement of caregivers (66%) may therefore help to explain these surprising results.

A positive correlation was also found between scores on the PSS’s ‘technical/professional activities’ dimension and healthcare professionals’ consideration of the specific needs of adults with intellectual disabilities. The persons with intellectual disabilities and their caregivers who took part in the study by Ali et al. (2013) also reported that satisfaction was influenced by consideration for patients’ specific needs. The present study only partially confirmed this, considering that the specific needs of adults with intellectual disabilities did not correlate with the PSS’s total score or the ‘educational relationship’ and ‘relationship’ dimension scores. Ali et al. (2013) included only people with mild, moderate intellectual disabilities which may potentially explain this difference in results. In this study, data were collected via a structured data collection developed for the propose of the study (Ali et al., 2013).

A negative correlation was observed between scores for the PSS’s ‘technical/professional activities’ dimension and the CAHS’s ‘damage/loss’ dimension. These results suggested that the more challenging the hospitalization, the less satisfied the patient was with the technical and professional interventions performed by their nurses. However, these results are incomparable because, to the best of our knowledge, there are no other quantitative studies on adults with intellectual disabilities’ cognitive appraisals of their hospitalizations. One possible explanation is that technical care is considered within the PSS’s ‘technical/professional activities’ dimension. Technical care is often invasive, which may explain why it is perceived as damaging to the person receiving it.

### The Interaction Model of Client Health Behavior

The links established in Cox’s Interaction Model of Client Health Behavior were not found in our study. Indeed, cognitive appraisal, sociodemographic, clinical, or hospitalization data had no influence on participants’ satisfaction scores. Cox (2003) nevertheless stated that health behaviors could be better explained by evaluating the effects of different variables simultaneously and interdependently; however, our small sample size did not allow this type of analysis, which may explain the lack of an established relationship. However, Carter and Kulbok (1995) evaluated the Cox model, noting that they found no relationship between client singularity and health outcomes, which may also explain our lack of an identified relationship. Our study did not directly assess client–professional interaction, which may explain why we did not find the links established in Cox’s Interaction Model of Client Health Behavior. However, the PSS indirectly assesses some components of the client–professional interaction (information and professional or technical skills) (Levandovski et al., 2015). Moreover, Carter and Kulbok (1995) noted that the Interaction Model of Client Health Behavior had never been tested in its entirety, and furthermore, it was not designed specifically for people with intellectual disabilities. The Interaction Model of Client Health Behavior, therefore, may not consider the specific needs of this patient group.

### Strengths and limitations

Among its strengths, this was the first study conducted in Switzerland to quantitatively evaluate adults with intellectual disabilities’ satisfaction with their hospitalization. It is also the first Swiss study to evaluate their cognitive appraisal of hospitalization and the factors associated with their satisfaction. Our study suggests that adults with intellectual disability can participate in quantitative studies, although it is preferable that the investigator also be present. The presence of a caregiver and breaks in the interview also facilitated the questionnaire’s completion.

The study recruitment process respected the principle of self-determination suggested for adults with intellectual disabilities (Schalock et al., 2021). It was also in line with the United Nations Convention on the Rights of Persons with Disabilities, whose objective is full participation of people with disabilities in society (United Nations & United Nations Human Rights, 2007).

Questionnaires were administered sometime after hospitalization, thus avoiding response bias (Polit and Beck, 2017).

Nevertheless, the study had some limitations. The measurement scales used have yet to be validated in French or in the type of population studied. In addition, the questions in the PSS refer to nurses (Rose, 2010); however, it is likely that the participants did not distinguish between nurses, nursing assistants, and doctors.

A social desirability bias is also likely (Polit and Beck, 2017), although this is possible to all satisfaction studies (Badejo et al., 2022). Satisfaction study typically have strong ceiling effects and limited variability (Badejo et al., 2022). Moreover, people are generally reluctant to criticize the care they receive (Santos de Almeida et al., 2015).

### Recommendations for practice, education, and research

At an acute care hospital level, policies can encourage approaches that are person centered to consider adults with intellectual disability needs, and explicitly focused on the inclusion of effective communications kills with this population. It would also be appropriate for hospitals to engage liaison nurses specialized in intellectual disability to improve adults with intellectual disabilities’ overall experience and satisfaction with their hospitalization. Indeed, various other studies have recommended establishing such nursing roles (MacArthur et al., 2015; Tuffrey-Wijne et al., 2014). These nurses’ roles would involve facilitating hospitalizations and limiting their potentially traumatic nature as much as possible. The specific support program for adults with intellectual disabilities developed at Geneva University Hospitals, which includes a specialized liaison nurse, has shown that this can enable adults in Switzerland with intellectual disabilities to have a better experience of hospitalization (Lalive d’Epinay Raemy and Paignon, 2019).

The more communication is centered on the patient, the better hospitalization is experienced by the adult with intellectual disability (Bernier Emch et al., 2021; Bradbury-Jones et al., 2013). At a practical level, it is important that healthcare professionals pay much attention to the relational aspects of care and strengthen a direct communication with adults with intellectual disability. Moreover, to increase adults with intellectual disabilities satisfaction, healthcare providers should use ways in which care delivery to this population could be improved (Iacono et al., 2014).

Both institutions and nursing educators could be proactive in training healthcare professionals. Thus, it would be interesting to develop continuing education with theoretical and practical training to provide healthcare professionals with extra knowledge and expertise on adults with intellectual disabilities, their care, needs, and health, communicating with them, and the importance of transmitting information to them. This would allow healthcare professionals to offer this population more holistic care, which should reduce their perceptions of the challenges facing them during their hospitalization and the health inequalities they experience (Mimmo et al., 2022; Schalock et al., 2021). It might also be relevant to integrate this knowledge into courses on the health and care of adults with intellectual disabilities and into the basic training of healthcare professionals.

In view of the cognitive appraisal difficulties encountered by people with intellectual disabilities during their hospitalization, it would be advisable to try to reduce their number. To do this, it would be relevant for medical and social institutions hosting adults with intellectual disabilities to make specific health assessments that take into account the problems they encounter, as it has been recommended by other studies (Casson et al., 2018; Robertson et al., 2014). These health assessments should enable the faster detection and management of their health problems and thus reduce hospitalizations and the challenges and losses they experience. To do this, nurses working in such institutions should be trained in clinical assessment, and health professionals working in these settings should be aware of the health problems that adults with intellectual disabilities frequently encounter. Making healthcare professionals aware of this population’s common health problems enables better detection of them. Reducing hospitalizations is consistent with current political, medical, social, and ethical thinking (Swiss Federal Office of Public Health, 2019).

Larger-scale research on adults with intellectual disabilities’ satisfaction and cognitive appraisal of hospital settings is needed for generalizable results. Research on the factors influencing satisfaction would help determine whether the relationships established in the Cox model hold true when analyzing different factors’ cumulative effects on satisfaction. It would also be pertinent to determine whether the factors of physical disability, limitations in the activities of daily living, illness severity, and type of hospital ward are associated with this population’s hospital satisfaction, as they are all potentially associated with it (Iacono and Davis, 2003). Whether caregivers can reliably transmit the correct level of satisfaction and cognitive appraisal of adults with intellectual disabilities, should also be assessed.

### Conclusion

This first quantitative study of adults in Switzerland with intellectual disabilities demonstrated that they were not fully satisfied with the care they received and had not had a very good experience with their hospitalization because they had encountered numerous challenges and losses. The study also showed that adults with intellectual disabilities were less satisfied with the care they received during hospitalization than were Switzerland’s GP, suggesting that Switzerland’s hospital system is not optimally equipped to meet their specific needs. Although this study’s results are not generalizable, they suggest that the hospital care provided to adults with intellectual disabilities in French-speaking Switzerland requires a rethink so as to offer them better and more specific support in hospital settings. This would require the development of health assessments specific to this population and targeted training for the healthcare professionals caring for them.
